# Infectious Etiology of Vomiting in Children With Presumed Acute Gastroenteritis in the Absence of Diarrhea: Protocol for a Cohort Study

**DOI:** 10.1097/PG9.0000000000000268

**Published:** 2022-10-25

**Authors:** Aleksandra Cepińska, Maciej Kołodziej, Edyta Podsiadły, Hania Szajewska

**Affiliations:** From the * Department of Paediatrics, The Medical University of Warsaw, Warsaw, Poland; † Department of Pharmaceutical Microbiology, Centre for Preclinical Research, Faculty of Pharmacy, The Medical University of Warsaw, Warsaw, Poland.

**Keywords:** pediatrics, enteropathogens, rectal swabs

## Abstract

NCT05270291, https://clinicaltrials.gov/ct2/show/

**Objectives:**

In children with acute gastroenteritis (AGE), vomiting often precedes diarrhea. To establish the diagnosis of AGE, enteropathogen detection typically relies on diarrheal stool samples. However, testing requires sufficient stool sample, which may not be easily available. Recent studies suggest that in children presenting to emergency departments with presumed AGE with isolated vomiting, an enteropathogen can be identified using rectal swabs and molecular diagnostic tests. The rate of enteropathogen detection in children with isolated vomiting due to AGE may differ in various populations. Using rectal swabs and molecular diagnostic tests, we plan to assess the proportion of children with isolated vomiting with presumed AGE in whom an enteropathogen can be identified.

**Methods:**

This will be a cohort study conducted in the emergency department(s) of one or more pediatric hospital(s) in Poland. Children younger than 5 years with the presence of ≥3 episodes of vomiting due to presumed AGE, lasting no longer than 7 days before enrollment, will be recruited. The primary outcome will be the proportion of children with isolated vomiting in whom an enteropathogen is detected. In all eligible participants, rectal swabs will be taken to perform molecular testing for detection of typical viral and bacterial enteropathogens. All children will be followed-up at 14 days after the initial contact to classify them into one of three groups (i.e., vomiting only, vomiting and diarrhea, and diarrhea only).

**What Is Known**

- Children presenting with isolated vomiting usually are not able to provide a suitable stool sample for pathogen testing.
- Due to the lack of alternative diagnostic methods, there is limited data about infectious etiologies of vomiting.

**What Is New**

- In Poland, the role of pathogens in AGE, may be influenced by the recent introduction of rotavirus vaccination and due to the influx of not vaccinated refugees from Ukraine.
- A recent Canadian study suggests, that in children with AGE with isolated vomiting, an enteropathogen can be identified using rectal swabs in nearly 50%.
- Presumably, this will be the first study in Poland using rectal swabs to assess the rate of enteropathogen identification in children with isolated vomiting.

## INTRODUCTION

Acute gastroenteritis (AGE) is defined as a decrease in the consistency of stools (loose or liquid) and an increase in the frequency of evacuations (typically ≥3 in 24 hours), with or without fever or vomiting (1). Worldwide, it remains one of the major causes of children’s morbidity and mortality (2). Compared with low- and medium-income countries, in high-income countries the mortality due to AGE is low; however, it remains a common cause of pediatric consultations and hospitalizations (3).

In children with AGE, vomiting often precedes diarrhea, especially in cases of rotavirus or norovirus infection (4). In countries that have introduced rotavirus vaccinations into their national immunization programs, a significant reduction in the number of children seeking medical attention for rotavirus infection was noted (5,6). Even if rotavirus remains the most common cause of AGE, the prevalence is low (7). Infections caused by other viruses, including norovirus, which is characterized by diarrhea and stomach pain, but especially by vomiting, remain unaffected by the introduction of the rotavirus vaccine (8). In Poland, rotavirus vaccination was introduced in January 2021 (9). Thus, a similar downward trend in the number of rotavirus infections was expected. However, since February 24, 2022, because of the war in Ukraine, more than 2 million people, mainly women with children, have crossed the Polish border. Such crises are always associated with an increased risk for gastrointestinal infections in children, which may be further increased as the rotavirus vaccine is not included in the routine Ukrainian vaccination schedule (10). Thus, in our setting, the role of various pathogens in AGE, including AGE with isolated vomiting, may be influenced by the current situation.

To establish the diagnosis of AGE, enteropathogen detection typically relies on diarrheal stool samples. However, children presenting with isolated vomiting usually are not able to provide a suitable stool sample for routine pathogen testing, and due to the lack of effective alternative diagnostic methods, there is limited data about infectious etiologies of vomiting in the absence of diarrhea (11–15). A recent study by Freedman et al (16) suggests that, in children presenting to emergency departments with presumed AGE with isolated vomiting, an enteropathogen can be identified using rectal swabs and molecular diagnostic tests. The implementation of these swabs and tests in patients with AGE presenting as isolated vomiting can improve patient management and reduce the use of antibiotics, diagnostic imaging, and endoscopy (17–20). Information about the detection rates and types of enteropathogens in children with isolated vomiting due to AGE remains limited and may differ in various populations.

### Study Objective

Using rectal swabs and molecular diagnostic tests, we plan to assess the proportion of children with presumed AGE and isolated vomiting presenting to the emergency department in whom an enteropathogen can be identified.

## METHODS AND ANALYSIS

### Design and Study Setting

This will be a cohort study. The recruitment will take place primarily in the emergency department and pediatric wards of the Pediatric Hospital of the Medical University of Warsaw, Warsaw, Poland. The recruitment is planned to start in May 2022 and last for 2 years. In case of a low recruitment rate, inclusion of outpatients and involvement of other recruiting wards and sites are under consideration provided that the personnel are adequately trained and competent in conducting clinical trials.

### Eligibility Criteria

#### Inclusion Criteria

Children eligible for this study must fulfill all of the following criteria:

– age 0–5-year-old
– presence of ≥3 episodes of vomiting due to presumed AGE at inclusion
– <7 days of symptoms at the time of inclusion
– signed written informed consent

#### Exclusion Criteria

– anticipated inability to complete 14 days of follow-up
– history of neutropenia (as rectal swabs are contraindicated)
– critically ill status requiring urgent medical intervention
– inability to provide a rectal swab for testing
– chronic gastrointestinal tract disorders such as inflammatory bowel disease, cystic fibrosis, celiac disease
– immunodeficiency

### Rationale for Age-group Selection

The study by Freedman et al (16) showed that over 90% of children with isolated vomiting presenting to emergency departments were less than 10 years of age. At the same time, children younger than 5 years of age have a higher risk of severe illness, dehydration, and hospitalization due to AGE. This age criteria allows us to assume that the population aged younger than 5 years will have the most clinical significance.

### Participant Timeline and Follow-up

For the study flow, see **Table 1**.

**TABLE 1.** Study flow

	Study period	
	Enrollment	Follow-up screen
Timepoint**	**Day 1**	**Day 14**
Enrollment		
Eligibility screen	X	
Informed consent	X	
Baseline information screen	X	
Rectal swab procedure	X	
Assessments		
Actual specimen testing for enteropathogens	As soon as possible after obtaining the rectal swab	
Symptoms developed after enrollment		X
Rectal swab result to caregiver via telephone/email		After receiving the result

The study investigator working at the study site will recruit participants among patients presenting to the emergency department and hospitalized due to vomiting with presumed AGE. All children aged 0–5 years potentially meeting the inclusion criteria will be screened for eligibility. After obtaining informed consent from the caregiver, study personnel will collect demographic and clinical data. A single rectal swab will be collected in the emergency department or pediatric department if the child is admitted to the hospital. Caregivers will be contacted again 14 days after enrollment via email and telephone, according to stated preference, to complete a survey regarding symptoms experienced during that interval and to identify any additional clinical care received.

### Group Classification

In line with Freedman et al (16), participants will be classified into one of three groups based on their symptoms: (1) isolated vomiting (i.e., no diarrhea); (2) isolated diarrhea (i.e., no vomiting); (3) mixed (i.e., vomiting and diarrhea). The data will be presented based on three time periods: (1) symptom onset until the hospital visit (defined as the emergency department or hospital visit); (2) time between hospital visit until the 14-day follow-up contact; (3) symptoms at time of 14th-day follow-up contact. Participants will be classified as positive for an enteropathogen if the rectal swab yields positive results. One exception will be *Clostridioides difficile* detection in children younger than 2 years of age. Data suggest that, in this age group, *C. difficile* is only exceptionally pathogenic (21).

### Data Collection

The following participant data will be collected at inclusion to the study and during the follow-up survey.

– Baseline participant characteristics, that is, age, gender, nationality (e.g., Polish, Ukrainian), any chronic illnesses, rotavirus vaccination status, antibiotic therapy in preceding 60 days.
– The characteristics of the presenting symptoms.
– If possible, an alternative diagnosis identified based on presenting symptoms and laboratory tests will be presented (e.g., urinary tract infection, bowel obstruction, appendicitis, pyloric stenosis, intussusception, gastrointestinal tract foreign body, neuroinfection, intracranial bleeding, intracranial malignancies).
– Tests performed, treatments provided, and disposition based on presenting symptoms (any stool test for pathogens, abdominal ultrasound, abdominal x-ray, head CT scan, urinalysis, urine culture, any blood tests, intravenous fluids administration, hospital admission, length of hospital stay).

### Specimen Testing

Rectal swabs will be collected by inserting the flocked swab through the rectal sphincter 1–1.5 inches (2–3 cm) and gently rotated (22), then it will be collected to 2 mL of Cary-Blair medium plastic tube. Specimens will be stored not more than 72 hours at 2–8°C before testing. Collection and transport will be performed per standard operating procedures. Stool samples will be tested for enteric viral and bacterial pathogens using BD Max Enteric Viral Panel (Max EVP) and BD MAXTX Enteric Bacterial Panel with the BD Max instrument (Becton Dickinson Co) according to the manufacturer’s instructions. The multiplex real-time PCR viral assay uses the primer and probe sets for detection of the following pathogens: Norovirus GI and GII, Rotavirus A, Adenovirus F40/41, Human Astrovirus, and Sapovirus genogroups I, II, IV, V. The bacterial PCR assay enables detection of *Salmonella* spp., *Shigella* spp., enteroinvasive *Escherichia* coli, *Campylobacter jejuni*/*Campylobacter coli,* and *Shiga* toxin-producing organisms. Primers and probe sequences are proprietary and not available for publication. The sample will be vortex to efficiently release any microbes associated with the swab before analysis. Then 50 µL of the sample will be placed in a tube containing diluent formulated to minimize inhibition associated with stool matrices. Each sample will be processed in individualized unitized reagent strips. Lysis and DNA extraction and PCR reaction will be automatically performed by the instrument. The specimen with a sample control processing (SCP) containing a plasmid with a proprietary synthetic target DNA sequence will be incorporated into the extraction tube to monitor effectiveness of DNA extraction, amplification and detection steps or reagent failures. Positive and negative controls are going to be included in each Max EVP run. A software algorithm within the BD Max instrument will interpret the amplification data and provide a positive or negative result. Only one repeat will be performed for each sample. Samples with a failed SCP result will be repeated three times. If the results of SPC is repeatedly negative, the specimen will be reported as unresolved.

### Unscheduled Visits

In case of an unscheduled visit, i.e., due to dehydration, the participant will receive a regular consultation in the emergency department. All the unscheduled visits will be recorded.

### Safety Management

Since the study procedure (i.e., rectal swab) does not involve greater than minimal risk to participants who meet the inclusion and exclusion criteria, adverse events are not expected. If any unanticipated problems related to the research involving risks to subjects or others happen during this study, these will be reported in a case report form (CRF).

### Data Collection, Data Management, and Confidentiality

We will create a coded, paper CRF containing all of the baseline information, symptoms, and history diaries for each patient. After completion of the study, the researcher will transfer data from the CRFs to an electronic (Excel) database. The collected data will be stored in areas with limited access. Confidentiality of data, including the personal data of the study participants, will be maintained at all times.

### Primary Outcome Measure

The primary outcome measure will be the proportion of children with isolated vomiting with presumed AGE in whom an enteropathogen is detected.

### Secondary Outcome Measures

– Types of pathogens detected based on presenting symptoms.
– The proportion of children with isolated vomiting with an alternative diagnosis.

### Sample Size

The primary outcome of this study is the proportion of children with isolated vomiting with presumed AGE in whom an enteropathogen can be identified. Assuming that 50% of the subjects in the study population have the factor of interest (17), the study would require a sample size of 165 for estimating the expected proportion with 5% absolute precision and 80% confidence. Predicting 20% lost to follow-up, a total sample size of 198 participants will be needed (23).

### Statistical Methods

We will use descriptive statistics to summarize baseline characteristics. We plan to calculate and present frequencies and percentages for categorical variables and medians and interquartile ranges (IQRs) for continuous variables. For data approximating a normal distribution, the Student t-test will be used to compare mean values of continuous variables. We will use the Mann-Whitney *U* test for non-normally distributed variables and the χ^2^ test or Fisher’s exact test, as appropriate, to compare percentages. We aim to calculate for continuous outcomes, differences in means or differences in medians (depending on the distribution of the data), and for dichotomous outcomes, the relative risk (RR), all with a 95% confidence interval (CI). The difference between study groups will be considered significant when the *P* value is <0.05, when the 95% CI for RR does not include 1.0, or when the 95% CI for mean difference (MD) does not include 0. Statistical significance will be based on two-tailed tests with a *P* value of 5%. We will present all estimates with a 95% CI.

### Patient Involvement

Patients were not involved in the creation of the study protocol.

### Research Checklist

We will use the STROBE guidelines when presenting the results in a manuscript (24).

## ETHICS AND DISSEMINATION

The study protocol was approved by the Bioethics Committee of the Medical University of Warsaw. In case of low recruitment, if new sites will be needed, additional bioethics approvals will be obtained.

### Protocol Amendments

Any modifications to the protocol that may affect the conduct of the study, represent a potential benefit for the patient, or may affect patient safety, including changes to the study objectives, study design, patient population, sample sizes, study procedures, or significant administrative aspects, will require a formal amendment to the protocol. Such an amendment will be submitted to the Bioethics Committee of The Medical University of Warsaw for approval and reported to the ClinicalTrails.gov platform.

### Consent

Before recruiting a patient into the study, the patient’s caregiver will receive from a trained team member information about the study, including the study’s objective, design and methodology, the possibility of adverse events, and possible risks and benefits of taking part in the study. The study information sheet, including the study’s main aspects and the investigator’s contact information, will be provided to the patient’s caregiver. The study team member will answer any questions regarding the study and will collect from the patient’s caregiver informed written consent.

### Dissemination Policy

The results of this study will be submitted for publication to a peer-reviewed medical journal and relevant national and international conferences. Information on completion of the study and its results will be indicated in ClinicalTrials.gov, where the study is registered. After completion of the study, patients’ caregivers who have expressed such a desire at the recruitment stage will receive information about the study results via email.
